# Risk stratification for lung adenocarcinoma on EGFR and TP53 mutation status, chemotherapy, and PD‐L1 immunotherapy

**DOI:** 10.1002/cam4.2492

**Published:** 2019-08-13

**Authors:** Chih‐Hsun Wu, Ming‐Jing Hwang

**Affiliations:** ^1^ Institute of Biomedical Sciences Academia Sinica Taipei Taiwan

**Keywords:** drug resistance, gene signature, lung cancer prognosis, target therapy, transcriptomics

## Abstract

The overall survival rates for lung cancer remain unsatisfactorily low, even for patients with biomarkers for which target therapies or immunotherapies are recommended. Better identification of at‐risk patients is needed to achieve more effective personalized treatment. Here, we derived a risk‐stratifying gene signature consisting of five genes that had the greatest differential expression by stage from lung adenocarcinoma (LUAD) transcriptomes. The new gene signature enabled survival prognosis for multiple LUAD datasets from different platforms of transcriptomics and risk stratification for patients with and without a mutation in *TP53* or *EGFR*, with high and low levels of *PD‐L1*, and with and without adjuvant chemotherapy treatment. Using these evaluations, it was also shown to be more robust compared to several other gene signatures. Functional analysis of the five genes and their protein‐protein interaction partners indicated that they are functionally enriched in cell cycle, endocytosis, and *EGFR* regulation, which are biological processes associated with lung cancer and drug resistance. Extensive discussions on related experimental studies suggest that the five genes are novel and sensible targets for developing new drugs and/or tackling drug resistance problems for LUAD.

## Graphical abstract

Advancing treatments for complex and deadly diseases needs gene signatures to identify patients at risk under various genetic and clinical conditions. Our new prognosis gene signature for lung adenocarcinoma meets that need with risk‐stratification ability on mutation status of *EGFR* and *TP53*, levels of immune checkpoint *PD‐L1* expression, and with and without adjuvant chemotherapy. This is a helpful step toward selecting an effective, personalized treatment plan for lung adenocarcinoma patients.

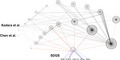

## 1 INTRODUCTION

Lung cancer is a leading cause of death for modern humans. Early detection allows complete surgical resection to offer the most effective treatment, but 40% of early‐stage patients relapse within 5 years of surgery. Adjuvant chemotherapies (ACTs), target therapies and immunotherapies have expanded treatment options, especially for nonsmall cell lung cancer (NSCLC).^1^ However, the 5‐year survival rate for patients with nonlocalized lung cancer remains dismal, with less than 30% for patients with regional disease and less than 5% for those with metastatic disease (https://seer.cancer.gov/statfacts/html/lungb.html).

Randomized trials have shown that patients with stage II or stage III lung cancer can benefit from ACT,^2^ and as such ACT following surgery is recommended for stage II‐III NSCLC patients by the National Comprehensive Cancer Network.^1^ However, current clinical staging and treatment guidelines cannot precisely distinguish between patients who would benefit from ACT and those who would not.

Target therapies require identifying patients whose cancer cells carry certain molecular markers that can be effectively targeted by specific drugs. For example, tyrosine kinase inhibitors (TKIs) such as osimertinib and erlotinib are typically used to treat patients with a TKI‐sensitive mutant epidermal growth factor receptor (*EGFR*). For patients with wild‐type *EGFR*, additional molecular tests are required to determine further treatments.^1^ But making treatment decisions based on *EGFR* mutation status is not straightforward because of the observations that not all patients with mutant *EGFR* experienced the expected better outcome with TKI treatment, and also that TKI treatment can help some patients with wild‐type *EGFR*,^3^ although there are reports to the contrary.^4^

*TP53* plays a pivotal role in regulating cancer development and is therefore a potential biomarker and drug target for cancer treatment. Various small‐molecule compounds can suppress the oncogenic functions of mutant *TP53* or restore the tumor suppressor activities of wild‐type *TP53*, and although they are not the current standard of care for NSCLC patients they are being tested in some clinical trials.^5^, ^6^ However, as for *EGFR*, effective treatments may require knowledge of the type of patients, with mutant or wild‐type *TP53*, who can benefit from the targeted therapy.

More recently, compounds that target immune checkpoints, such as the programmed death‐1 (PD‐1) or programmed death ligand‐1 (PD‐L1),^7^ are infusing optimism to the fight against cancers. For example, Pembrolizumab, a type of anti‐PD‐1 immunotherapy drug, is recommended for treating advanced patients without a mutation in *EGFR* and anaplastic lymphoma kinase (*ALK*) and with high *PD‐L1* expression.^1^ But for NSCLC at least, while durable responses have been observed in some patients, checkpoint inhibition does not have the same effect for all the patients.^7^ Additional biomarkers besides *PD‐L1* expression are needed to achieve improved efficacy of immunotherapies.

These results and the fact that chemotherapies, target therapies, and immunotherapies can cause serious adverse effects provide an impetus to advance precision medicine. To that end, prognostic biomarkers for identifying at‐risk patients under different genetic and clinical conditions are urgently needed to enable effective while avoiding ineffective, potentially harmful, therapies.

Most cancer prognostic biomarkers, including those for lung cancer, are genes with a prognostic expression profile. These so‐called gene signatures (GSs) are usually derived by correlating patients’ survival data with gene expression data through Cox proportional hazards modeling.^8^ Many such GSs for lung cancer have been reported,^9^ but most have not been examined for their prognostic effectiveness with regard to the aforementioned ACT, *TP53* and *EGFR* mutation status, and *PD‐L1* expression, or have been examined for one or more but not all of these conditions.

**Figure 1:**
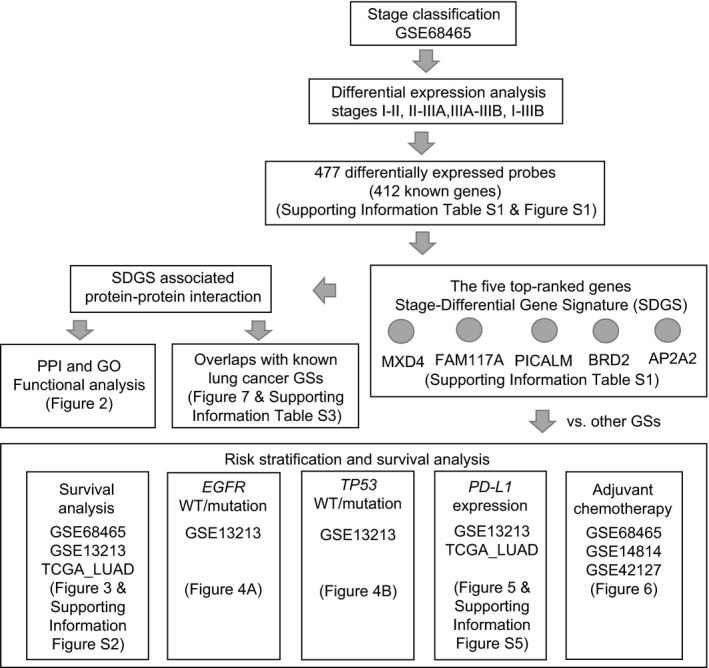
Flowchart of this study. SDGS was derived from analyzing stage‐dependent gene expression data of 443 LUAD patients from dataset GSE68465. The five top‐ranked differentially expressed genes were chosen to form SDGS, which was then undergone PPI and GO functional analysis and comparisons to several known lung cancer GSs on survival prognosis and risk stratification. The various datasets used in these analyses and the figures/tables in which the results are presented are indicated

Here, we report a new GS for LUAD that can identify at‐risk patients for all these conditions. The new GS is composed of five genes and is referred to as SDGS owing to the use of Stage‐Differential gene expression in its derivation. Unlike most other GSs, SDGS was not derived from patients’ survival data; such data merely served to test the signature's prognostic power. Below, we describe the derivation of SDGS, functional analyses of its five constituent genes, and stratification of at‐risk patients based on SDGS vs several other lung cancer GSs (see Figure 1 for a flowchart of this study). SDGS represents a novel prognostic GS for lung cancer. To the best of our knowledge, none of its five genes has appeared in previously identified lung cancer GSs. The five genes of SDGS are therefore potential novel targets for developing new target therapy drugs against LUAD.

## 2 MATERIALS AND METHODS

### 2.1 Datasets and stage‐differential gene expression

SDGS was derived from GSE68465, a gene expression dataset of 443 patients with LUAD (often denoted as ADC), with clinical and pathological annotations from the National Cancer Institute Director's Challenge Consortium for the Molecular Cancer of Lung Adenocarcinoma.^10^ This dataset and others used in this study for survival analysis (see Figure 1) are all freely available at SurvExpress (http://23.96.106.14:8080/Biomatec/SurvivaX.jsp) and Gene Expression Omnibus (GEO; https://www.ncbi.nlm.nih.gov/geo/).

Based on clinical information for the state of tumor (T), node (N), and metastasis (M), the 443 patients of GSE68465 were classified into stages I (T1‐T2 N0 M0), II (T1‐T2 N1 M0), IIIA (T3 N0‐1 M0, T1‐3 N2 M0), IIIB (any T4 or any N3 M0), and IV (any M1) according to Harrison's Principles of Internal Medicine.^11^ Differentially expressed microarray gene probes, hence genes through mapping, were identified by running R (http://cran.r-project.org) package ‘limma’ based on the *t*‐statistic between two different stages. Specifically, in this study, differentiation was between stages I and II, II and IIIA, IIIA and IIIB, and, to complete a loop, between I and IIIB. (There were no stage IV patients in GSE68465.) Adjusted *P*‐values of the *F*‐statistic to account for the Benjamini‐Hochberg false discovery rate were then used to rank differentially expressed genes. SDGS is composed of the five most differentially expressed genes. All statistical tests in this study are two‐sided.

### 2.2 Survival analysis and risk group stratification

Using the ‘coxph’ function in the ‘survival’ package of R, patients’ survival data were regressed against their expression levels of the five genes of SDGS to derive a Cox proportional hazards model.^8^ With this model, a survival risk score, could be computed for each patient as follows: PI (prognostic index) = β_1_x_1_ + β_2_x_2_ +… + β_5_x_5_, where β*_i_* is the coefficient of the Cox model for the *i*th gene, and x*_i_* is a value indicating its expression level for the patient. The patients were then ordered by their PI values and split into two groups by the median of the ordered PI; that is, patients in the high‐risk group had a PI higher than the median and those in the low‐risk group had a PI that was equal or lower than the median.

The performance of the resulting Cox model was evaluated by C‐index, a measure of concordance between predicted and actual survival status for any two patients. C‐index ranges from 0 to 1, with 1 being perfect prediction and 0.5 being equivalent to a random guess. In addition, the survival probabilities of patients from the date of the trial start until the last follow‐up contact or death were investigated by drawing the Kaplan‐Meier survival curve for both the high‐ and the low‐risk groups, or for any two groups, such as those who received ACT and those who did not. The *P*‐values of log‐rank tests were calculated to compare the survival differences between two groups.

In addition to GSE68465, SDGS‐based survival prognosis was evaluated for another GEO dataset (GSE13213) as well as RNA sequencing data of LUAD from the Cancer Genome Atlas (TCGA, https://cancergenome.nih.gov/), which we refer to as TCGA_LUAD hereafter (see Table S2 for a summary of all datasets analyzed in this study). Additionally, the ability of SDGS to predict survival probabilities for high‐ and low‐risk patients with or without a mutation in *TP53* or *EGFR* was evaluated with GSE13213, the dataset of LUAD patients with annotations of the mutation status for both genes. The ability of SDGS to predict the survival for LUAD patients with high or low expression level of *PD‐L1* was assessed for datasets GSE13213 and TCGA_LUAD in which information of *PD‐L1* expression was available. In this evaluation, patients were split into high‐expression and low‐expression groups by the median of the *PD‐L1* expression levels. Finally, given their inclusion of ACT status, GSE68465 and two additional GEO datasets (GSE14814 and GSE42127) were used to evaluate the ability of SDGS to identify LUAD patients who could or could not benefit from ACT. For comparison purposes, these evaluations were also carried out where relevant for two other five‐gene GSs^12^, ^13^ and a 12‐gene GS^14^ reported in the literature. The same procedures described above were followed to derive the Cox models to evaluate these other GSs.

### 2.3 Functional analysis

To investigate whether and how the five genes of SDGS might be related to lung cancer, we first retrieved their interacting proteins from a recently reported protein‐protein interaction (PPI) dataset^15^ that was compiled from integrating widely used PPI databases. The set composed of SDGS s five genes and their PPI partners was then subjected to gene ontology (GO) enrichment analysis on GO terms of Biological Process using the Cytoscape (https://cytoscape.org/) plug‐in ClueGO. The enriched functions (adjusted *P*‐value < .05) were connected into networks for visual inspection of their relationships.

## 3 RESULTS

### 3.1 Derivation of SDGS

As described in Materials and Methods, SDGS was derived by finding genes that were differentially expressed in different stages of GSE68465 patients who have been annotated with LUAD stage information. The analysis resulted in 477 differentially expressed probes (for 13 unknown genes and 412 known genes) with an adjusted *P*‐value of the *F*‐statistic < 0.05, and these are provided in Table S1. The top five differentially expressed genes ranked by *P*‐value of significance, *MXD4*, *FAM117A*, *PICALM*, *BRD2*, and *AP2A2*, were chosen to form SDGS by considering the balance between the number of top‐ranked genes selected and the resulting prognostic power, as shown in Figure S1.

### 3.2 Functions of the five genes of SDGS

We surveyed the literature to gather what is known about the functions of the five SDGS genes, particularly in relation to cancer. As described below, we found evidence to suggest that all five genes are likely associated with tumorigenesis or tumor progression, and some specifically with lung cancer.

MXD4, also called MAD4, is a member of the MAD family, which forms a transcriptional repression complex with MAX to increase cell differentiation and prevent proliferation.^16^ MXD4 can thus antagonize the oncoprotein MYC, which also interacts with MAX but with consequent induction of cell proliferation and tumorigenesis.^16^

FAM117A is a C/EBP‐induced protein.^17^ A recent study identified different populations of macrophages/monocytes from tumors at distinct stages of progression in a model of murine lung cancer.^18^ In that study, *FAM117A* was one of 2458 differentially expressed genes identified from pairwise comparisons made at various time points and between separate cell categories. FAM117A may therefore be associated with progression of lung cancer.

PICALM, phosphatidylinositol binding clathrin assembly protein, is also called CALM for clathrin assembly lymphoid myeloid leukemia protein.^19^ Somatic mutations of the splicing factor *U2AF1* are significantly associated with 30 RNA splicing alterations common in both acute myeloid leukemia and LUAD, and *PICALM* is among the genes found in those splicing alterations.^20^ Single‐nucleotide polymorphisms in *PICALM* are related to calcium channel blocker responses.^21^ Since calcium signaling is associated with tumorigenesis, angiogenesis, and metastasis of cancer cells,^22^ PICALM is a potential anticancer target. Indeed, *PICALM* is listed in the Cancer Gene Census, a gene mutation database of cancers.^20^

BRD2 is a member of the bromodomains and extra‐terminal domain (BET) family, which interacts with acetylated chromatin and transcription complexes to control transcription, and can bind MYC to drive tumorigenesis in lung cancer.^23^ BRD2 interacts with Runx3 to form a complex, and inactivation of Runx3 is an important early event in the development of LUAD.^24^

AP2A2 (adaptor‐related protein complex 2 (AP2) alpha 2 subunit) positively controls hematopoietic stem cells for asymmetric segregation.^25^ Interestingly, activation of *TP53* also increases asymmetric division in breast cancer stem cells.^26^ Functioning like TP53 to influence the fate of cancer stem cells, AP2A2 may likewise play a tumor suppression role.

Among the five genes of SDGS, the functions of *PICALM* and *AP2A2* are evidently linked. PICALM recruits AP2 and clathrin to cell membranes at the sites of coated‐pit formation to induce AP2‐dependent clathrin‐mediated endocytosis and clathrin‐vesicle assembly, thereby regulating cell proliferation and survival.^27^ AP2 and clathrin form a complex to interact with EGFR and affect endocytic uptake.^28^ This function can explain the finding that treatment effectiveness of erlotinib‐resistant cells was positively correlated with the expression of clathrin‐associated AP2 proteins, including AP2A1, AP2A2, and AP2B1.^29^ These findings may suggest a role of PICALM and AP2A2 in tumorigenesis and/or tumor progression.

**Figure 2:**
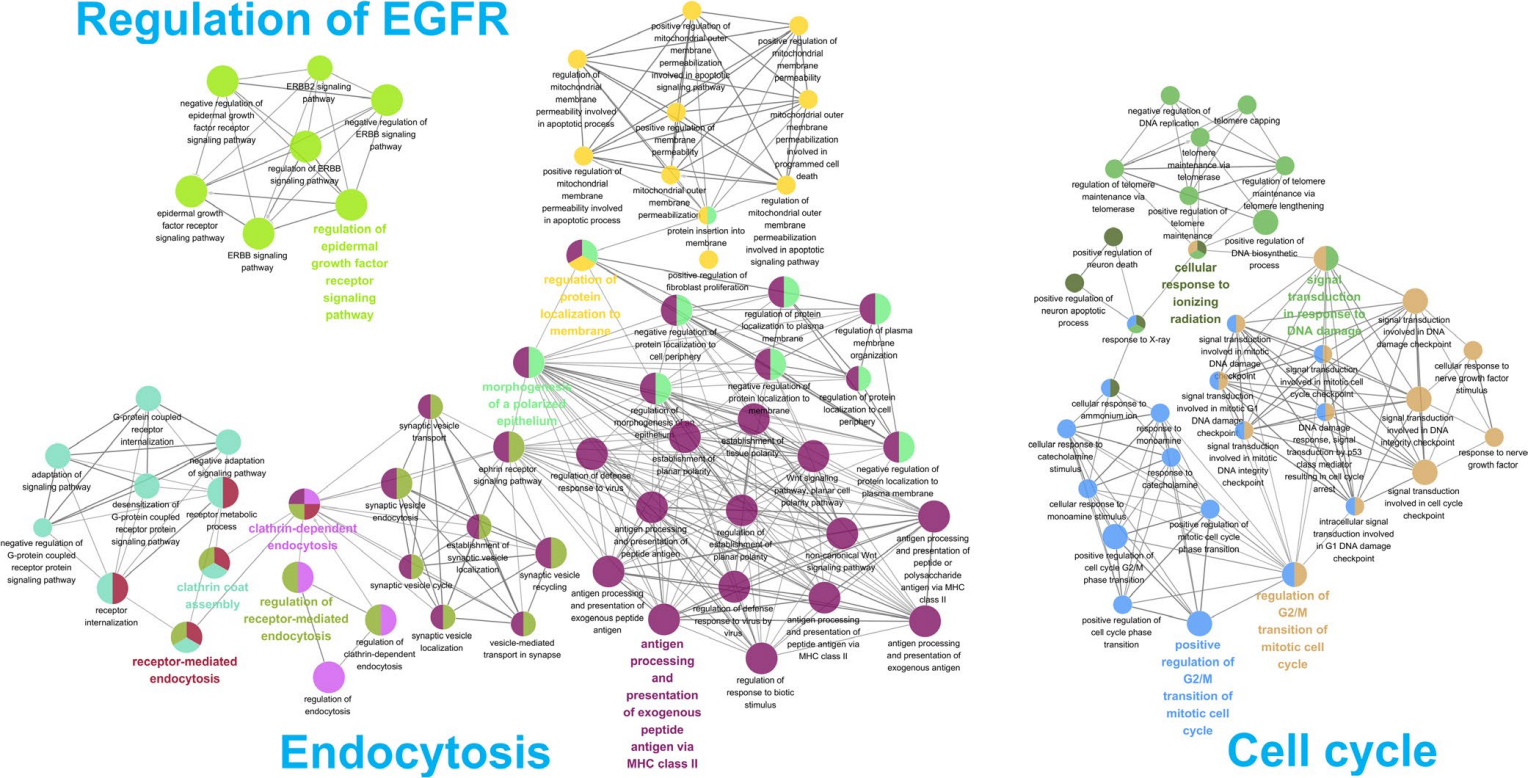
Functional characterization of SDGS genes and their protein‐protein interaction partners. In these networks of related functions, the nodes represent the enriched functions (GO terms of Biological Process) for the set of the five SDGS genes and their PPI partners (see Materials and Methods), and lines indicate the connected GO terms that showed up for the same gene or genes. Different enriched functions are encoded by different colors, and node size is scaled roughly to the level of enrichment significance (adjusted *P*‐values < .05). Multi‐colored nodes are those connected to more than one functions. Note that the analysis resulted in three large connected networks, whose functions can be generalized into three main functional categories, namely, regulation of EGFR, cell cycle, and endocytosis

Proteins usually interact with other proteins. Functional modules can thus be deduced from PPI networks, which are useful for studying cancer genes.^15^ We retrieved 68 interacting proteins for four of the five SDGS genes (no PPI information for the fifth, *FAM117A*, in PPI databases). Analysis of the GO terms of Biological Process for the five SDGS genes and their 68 PPI partners revealed three main networks connecting the enriched functional terms. These networks respectively included three major functional categories: cell cycle, endocytosis, and regulation of *EGFR* (Figure 2). These generalized functional categories are in accord with the specific functions extracted from the literature for the five SDGS genes.

### 3.3 Survival prognosis with comparison to other five‐gene GSs

Two five‐gene GSs for lung cancer are described in the literature. Chen et al derived a GS from a microarray analysis of 672 genes based on the correlation between their expression and survival data of 125 NSCLC patients. The analysis initially led to a 16‐gene GS and then, aided by further RT‐PCR analysis, the five‐gene GS.^12^ Kadara et al identified 584 genes that were differentially and progressively expressed within cells of a human in vitro lung carcinogenesis model. From those genes and an analysis of functional pathways, they derived a six‐gene GS that was later reduced to the five‐gene GS for LUAD prognosis, using data from the GSE68465 dataset.^13^

**Figure 3:**
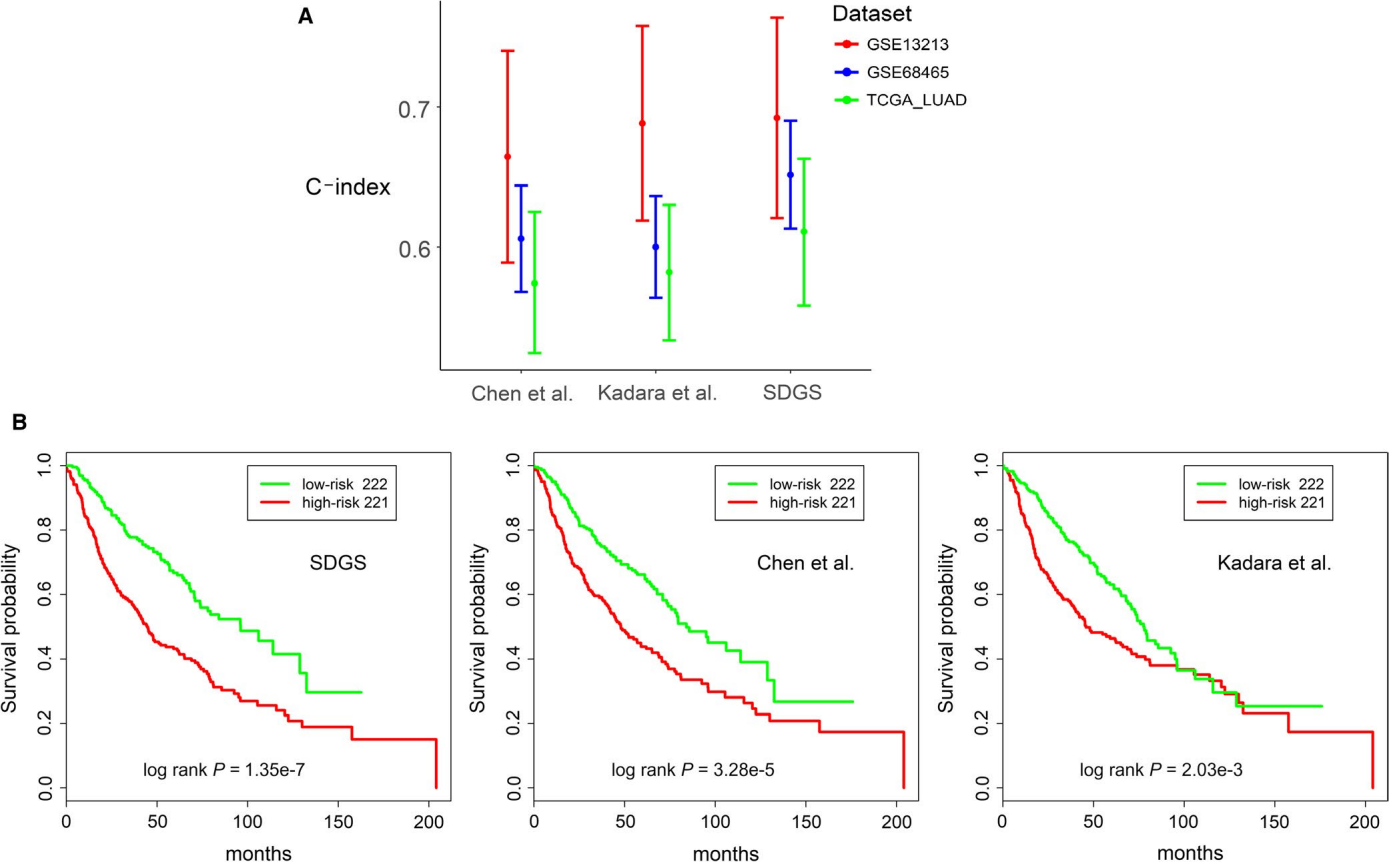
The prognostic performance of SDGS in comparison with two five‐gene GSs. A, C‐index results for three independent datasets (GSE68465, GSE13213, and TCGA_LUAD) by three five‐gene GSs. Error bar indicates 95% confidence interval. B, Kaplan‐Meier survival curves for low‐risk (green) and high‐risk (red) LUAD patients of dataset GSE68465 (see Figure S2 for the same analysis on datasets GSE13213 and TCGA_LUAD)

As shown in Figure 3A, for the three datasets evaluated (GSE68465, GSE13213, and TCGA_LUAD), the three five‐gene GSs were mostly comparable to each other with regard to their performance for survival prognosis as measured by C‐index. SDGS appeared to be more consistent, however, and slightly better than the other two GSs for datasets GSE68465 and TCGA_LUAD. The C‐index values achieved were generally around 0.65, which is similar to those reported in a benchmark assessment on multiple tumor types using a large set of diverse genomic and proteomic molecular data.^30^ The three GSs had comparable ability to stratify high‐ and low‐risk LUAD patients in these three datasets, as indicated by Figure 3B for the results on GSE68465 and Figure S2 for the results on GSE13213 and TCGA_LUAD.

### 3.4 Stratifying at‐risk patients with and without EGFR or TP53 mutation

**Figure 4:**
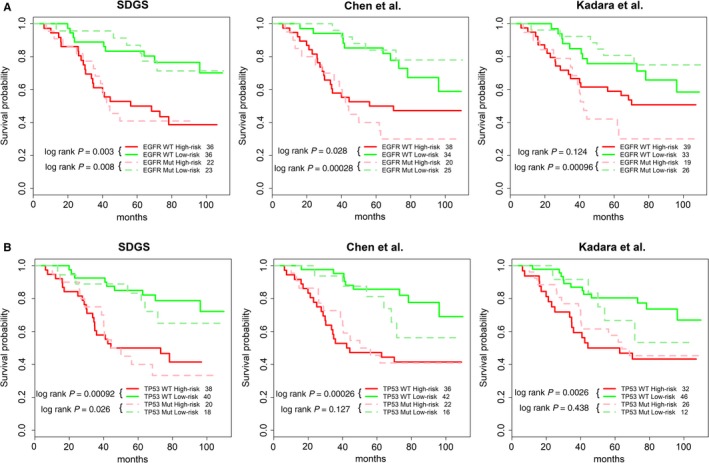
Survival probabilities of high‐risk and low‐risk LUAD patients with and without *EGFR* and *TP53* mutation by three five‐gene GSs. A, The 117 LUAD patients of dataset GSE13213 were divided into 58 high‐risk (red) and 59 low‐risk (green) patients based on their survival risk score computed by the respective Cox model of the three five‐gene GSs (see Materials and Methods, Figure 3B and Figure S2). Among those patients, 72 cases were *EGFR*‐WT and 45 were *EGFR*‐mutant. The patients’ survival probabilities in both the high‐risk and low‐risk groups were analyzed by Kaplan‐Meier curves to evaluate whether a statistically significant difference existed in the survival outcome between *EGFR*‐WT and *EGFR*‐mutant cases. B, Same as in A but for *TP53*, and among the 116 GSE13213 LUAD patients (removing one low‐risk patient with no information of *TP53* mutation status), 78 cases were *TP53*‐WT and 38 *TP53*‐mutant

*EGFR* mutation is a marker for target therapy of NSCLC, but its prognostic value is debated.^31^ We, and others,^31^ found that for the LUAD patients of GSE13213 who did not receive ACT, their overall survival outcomes were similar whether or not their *EGFR* harbored a mutation (Figure S3A). Nevertheless, identifying at‐risk patients of either *EGFR* wild type or mutation can facilitate clinical decisions on differential treatment plans. As shown in Figure 4A, all three five‐gene GSs generally possessed this ability, although the GS of Kadara et al^13^ was not as capable as SDGS or the GS of Chen et al^12^ of stratifying patients with wild‐type *EGFR* because its survival model could not separate the high‐risk and low‐risk patients with statistical significance.

Similarly, the *TP53* mutation had no significant effect on overall survival for lung cancer^32^ (see Figure S3B for GSE13213 patients). However, it was encouraging that SDGS could consistently identify at‐risk patients with or without a *TP53* mutation. In comparison, the other two GSs did not distinguish for patients with a *TP53* mutation with statistical significance (log rank *P* < .05; Figure 4B). Identifying at‐risk patients with and without a *TP53* mutation could help interpret clinical trial results of TP53 target therapies.

Although some studies have shown that some patients with wild‐type *EGFR* could benefit from TKI target therapy,^3^ other studies have indicated otherwise for certain subgroup of patients.^4^ Among such patients at an advanced stage, those without *ALK* mutation and with high *PD‐L1* expression can be treated with anti‐PD‐L1 immunotherapy.^1^ The patients with wild‐type *EGFR* from the high‐risk group that can be identified by SDGS would be good candidates for further tests and consideration of appropriate therapies.

### 3.5 Stratifying at‐risk patients with low and high PD‐L1 expression

**Figure 5:**
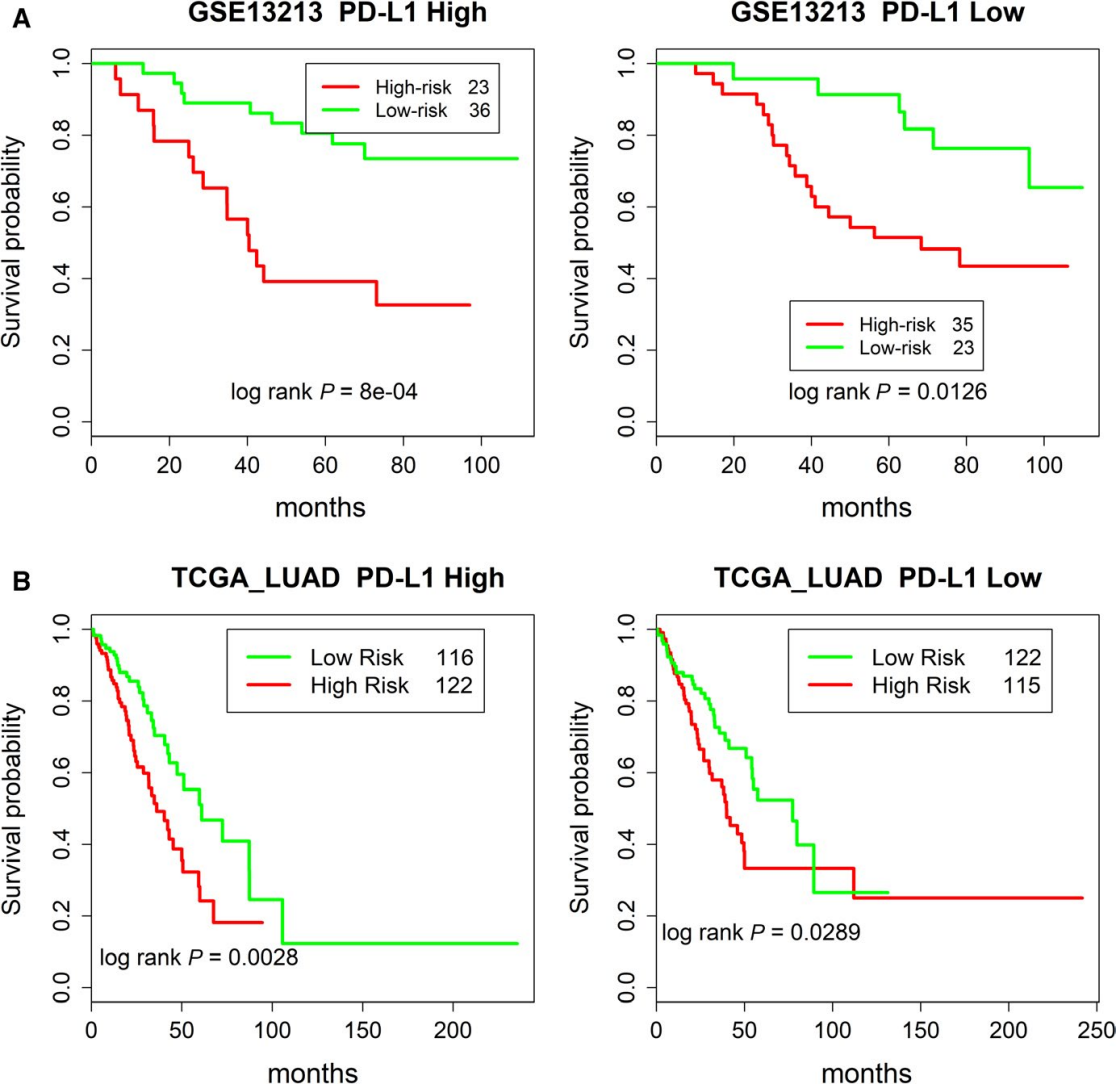
Survival probabilities of high‐risk and low‐risk LUAD patients with high and low *PD‐L1* expression for two different datasets by SDGS. Same as in Figure 4A by the Cox model of the SDGS but for high and low *PD‐L1* expression patient groups. A, Results for dataset GSE13213. B, Results for dataset TCGA_LUAD. See Figure S5 for the performances of two other five‐gene GSs

The overall survival probabilities of LUAD patients with low or high level of *PD‐L1* expression appear to be similar^33^ (also see Figure S4). However, as shown in Figure 5, SDGS is capable of separating high‐ and low‐risk patients from both groups of *PD‐L1* expression for LUAD patients of two different datasets, GSE13213 and TCGA_LUAD. Further, the risk‐stratifying ability of SDGS for LUAD patients with respect to *PD‐L1* expression was better than the other two GSs compared (Figure S5). The ability to identify high‐, as well as low‐risk patients in these conditions can be very helpful in making immunotherapy recommendations.

### 3.6 Stratifying at‐risk patients for ACT

Most GSs reported for lung cancer are used for prognosis only and have not been evaluated on whether a patient would benefit from ACT, which is often recommended to supplement surgical resection.^1^

**Figure 6:**
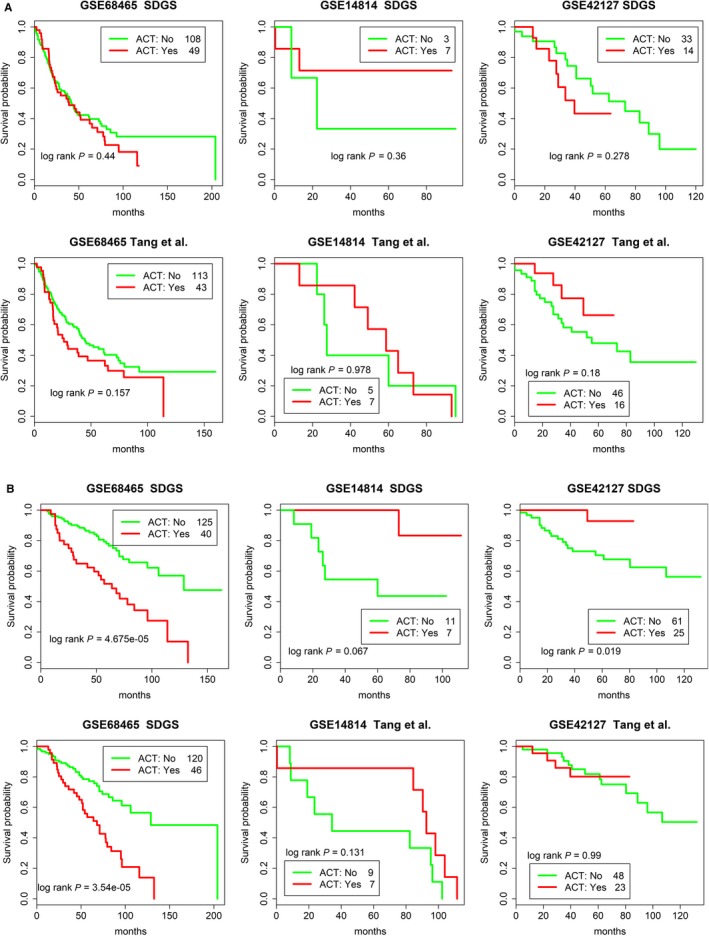
Survival probabilities of high‐risk and low‐risk LUAD patients with and without adjuvant chemotherapy (ACT) in three different datasets by SDGS and a 12‐gene GS. Same as in Figure 4A but for with (yes) and without (no) ACT, and the comparison to SDGS was made with a 12‐gene GS^14^ Cox model derived using the procedures described in Materials and Methods. In addition to GSE68465, patients of GSE14814 and GSE42127 with ACT status were analyzed. A, High‐risk group; B, Low‐risk group

Figure 6A shows that for high‐risk patients, SDGS could not distinguish the survival probabilities between patients who received ACT and those who did not for the three datasets (GSE68465, GSE14814, and GSE42127) that included data on LUAD patients and any ACT they had received. For low‐risk patients, however, results of the SDGS model indicated that those receiving ACT fared better in datasets GSE14814 (log rank *P* = .067) and GSE42127 (log rank *P* = .019), but worse in dataset GSE68465 (log rank *P* < .001) (Figure 6B). Largely similar results were obtained (Figure 6A,B) when the same modeling procedures and evaluations (see Materials and Methods) were applied to a previously reported 12‐gene GS,^14^ except that the 12‐gene GS model showed no significant ACT benefit for low‐risk patients in datasets GSE14814 (log rank *P* = .131) and GSE42127 (log rank *P* = .99). However, as reported by Tang et al,^14^ who used a somewhat different statistical model (a supervised principal component analysis model) for the 12‐gene GS, ACT could benefit high‐risk patients with LUAD and lung squamous cell carcinoma (LUSC) in these two datasets. Using a 94‐gene malignancy‐risk GS, another study also reported that high‐risk patients in dataset GSE14814 can benefit from ACT, but this GS did not predict an ACT benefit for low‐risk patients in the same dataset.^34^ For the GSE68465 dataset, results of the 94‐gene GS were similar to those shown in Figure 6A,B; that is, ACT was associated with a worse outcome for low‐risk patients and there was no difference for high‐risk patients.^34^

These results may appear confusing because different statistical models and different patients were used in these studies (eg, our analysis included only LUAD patients, whereas Tang et al^14^ and Chen et al^34^ analyzed both LUAD and LUSC patients). Nevertheless, with the exclusion of GSE68465, it seems that ACT can benefit a certain portion of both low‐risk (Figure 6B, for SDGS) and high‐risk^14^, ^34^ patients with LUAD. The seemingly opposite—that is, harmful effects of ACT for GSE68465 patients in both low‐risk (Figure 6B, for both SDGS and the 12‐gene GS) and high‐risk^34^ groups—is likely due to GSE68465 patients (all LUAD) exhibiting a poorer survival rate than the LUAD patients in the GSE42127 and GSE14814 datasets (Figure S6), despite patients’ staging data in these different datasets being not significantly different overall. Notably, 76% of GSE68465 patients smoked in the past and an additional 8% of patients were current smokers, suggesting a possible reason for their poorer survival rates.

## 4 DISCUSSION

Staging is a key clinical indicator of the progression of lung cancer and patients’ survival prospects. We showed that stage‐dependent gene expression data can be used to derive a very good prognostic GS for LUAD patients. In comparison with two other five‐gene GSs, the SDGS exhibited a more consistent survival prognosis for multiple independent datasets (Figure 3), as well as better performance in stratifying at‐risk patients under both low and high *PD‐L1* expression (Figure 5), and with and without *EGFR* and *TP53* mutations in both high‐ and low‐risk groups (Figure 4). Several other GSs have shown a similar *EGFR* and/or *TP53* stratifying prognosis,^35^, ^36^ and risk stratification for ACT has also been studied with other GSs,^14^, ^34^, ^37^ but SDGS was additionally capable of identifying at‐risk LUAD patients with low and high *PD‐L1* expression (Figure 5) as well as low‐risk patients who might benefit from ACT (Figure 6).

**Figure 7:**
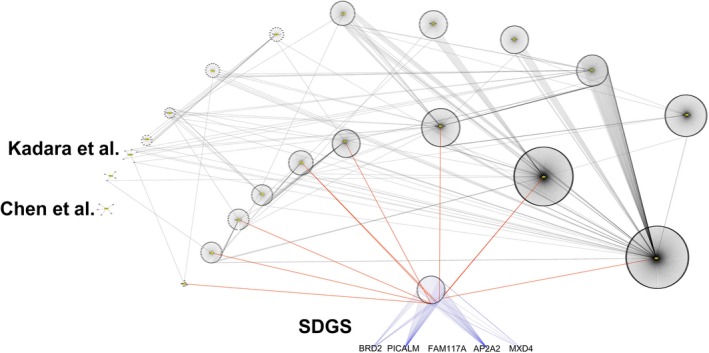
A network of lung cancer GSs showing limited overlap. Each dot is a gene belonging to a GS, depicted as a circled cluster, except for SDGS, which has its five constituent genes explicitly labeled, with each gene connected to its PPI partners shown in a circle (note that *FAM117A* has no PPI information as mentioned in the main text). As shown, none of the five genes of SDGS is in any of the known lung cancer GSs compiled by Tang et al,^9^ and just few (9) of their 68 PPI partners are included (connected by red lines) in some of those GSs. A line connects two identical genes appearing in two different clusters, but for visual clarity, lines are purposely converged at one end toward the center of the larger cluster. Quantitation of the overlap (ie, shared genes between any two GSs) is given in Table S3

SDGS is a novel GS for LUAD. To our knowledge, no other lung cancer GS has used any of its five genes; furthermore, only 9 of its 68 known PPI partners have been included in the other GSs to provide a prognosis for lung cancer^9^ (Figure 7; Table S3). However, given their functional activities (see Results) and the results of GO enrichment analysis (Figure 2), it is evident that the five genes of SDGS are lung cancer related.

In particular, of the five genes of SDGS, *PICALM*, *AP2A2*, and *BRD2* are known to be involved in *EGFR* regulation and/or resistance. PICALM and AP2A2 interact with EGFR,^28^ and AP2 family is associated with EGFR resistance to the TKI erlotinib through dysregulation of the endocytosis machinery.^29^ PICALM and AP2A2 are associated with clathrin and are thus closely involved in endocytosis, one of the three main functions enriched in SDGS genes and their PPI partners (Figure 2). EGFR endocytosis can be a pathway from which to find novel therapeutic targets for lung cancer with wild‐type EGFR.^38^ An abnormality in bypass signaling pathways is another of several mechanisms of EGFR resistance to TKIs,^39^ and one of those bypass pathways involves vascular endothelial growth factor (VEGF) and its receptor, VEGFR. BRD2 expression in endothelial cells was found to increase under VEGF stimulation, and inhibition of BRD2 repressed VEGF‐induced cell migration, angiogenesis, and proliferation.^40^ These studies suggest BRD2 could be a target to battle EGFR resistance to TKIs. Inhibition of BET proteins also increases the clinical efficacy of PI3K inhibitors.^23^ Since BRD2 is in a downstream pathway of EGFR signaling, its inhibition could affect EGFR resistance to TKIs. Interestingly, a PI3K inhibitor LY94002 not only blocks PI3K activity but also inhibits BET proteins BRD2‐4,^41^ suggesting that BRD2 could potentially have combined effects on EGFR resistance. Consistent with this notion, the combination of BET bromodomain inhibitor JQ1 and TKIs has been suggested to be a rational strategy for treating leukemia and lymphoma.^42^ Additionally, BRD2 was shown to positively control epithelial‐mesenchymal transition in breast cancer,^43^ and a GS for this histologic transformation could forecast the resistance to EGFR inhibitor erlotinib in both wild‐type *EGFR* and mutant *EGFR* lung cancer cases.^44^

SDGS also has three genes, *MXD4*, *BRD2*, and *AP2A2* that are known to be associated with *TP53*. As described in Results, the association of MXD4 and BRD2 with TP53 is through direct or indirect interaction with the MYC family,^16^, ^23^ which is influenced by *TP53* mutation.^45^ In addition, both AP2A2 and TP53 enhance the asymmetric segregation of cancer stem cells.^25^

EGFR and TP53 are related to PD‐1 and PD‐L1. EGFR can upregulate the PD‐L1 pathway to control immune escape,^7^ and the combination of PD‐1/PD‐L1 inhibitors and EGFR TKIs is a good strategy to treat NSCLC with *EGFR‐*activating mutations.^7^ *RAS/TP53* mutations are more constantly found in NSCLC patients who showed *PD‐L1* expression, which may provide a means to predict clinical efficacy of PD‐1/PD‐L1 inhibitors.^46^ Indeed, *TP53* and *EGFR* mutations are strong parameters to predict responses to anti‐PD‐1 treatment in NSCLS.^47^

There is also a connection between *PD‐1* and *PD‐L1* and at least two of the five SDGS genes, *PICALM* and *BRD2*. *PD‐L1* was expressed in *RET* (rearranged during transfection)‐rearranged NSCLC,^48^ and kinase fusions, such as the fusion of *PICALM* and *RET*, are associated with tumorigenesis.^48^ Inhibition of BRD2, which has a similar effect on STAT5, is an appealing therapeutic strategy for hematologic malignancies,^42^ consistent with the finding that enhanced STAT5 phosphorylation can increase *PD‐L1* expression to produce PD‐L1–mediated immune escape.^49^ Inhibition of both BET bromodomain (eg, BRD2) and immune checkpoints (eg, PD‐1) is also a promising approach to treat solid tumors such as LUAD.^50^

*FAM117A*, the remaining SDGS gene not discussed above, has not yet been associated with EGFR or TP53 or immune checkpoints and is less studied; however, a recent report implicated it as having a role in lung cancer, albeit in a mouse model.^18^

In conclusion, given all available evidence, our analysis suggests that SDGS can be more than just a prognostic biomarker for LUAD, and that the five genes of SDGS could provide ample possibilities in the development of new strategies for treating LUAD patients of different conditions relating to adjuvant chemotherapy, target therapy and immunotherapy.

## CONFLICT OF INTEREST

None declared.

## AUTHOR CONTRIBUTIONS

CHW and MJH conceived and designed the study. CHW carried out data collection and all computations. CHW and MJH analyzed the results and wrote the manuscript.
