# Supplementary material for: Risk stratification for lung adenocarcinoma on EGFR and TP53 mutation status, chemotherapy, and PD‐L1 immunotherapy
Source: Cancer Med. 2019 Aug 13;8(13):5850–61. doi: 10.1002/cam4.2492 (PMC6792489; doi:10.1002/cam4.2492)
Supplement: Supplementary file 2 [file CAM4-8-5850-s002.doc]

**Supporting Information Tables (S2 and S3) and Figures (S1-S6)**

**(**Note that **Supporting Information Table S1- The 477 differentially expressed probes** is presented in an excel file separate from this file)

**Supporting Information Table S2- Datasets and usage in this study**

**Supporting Information Table S3- Overlaps of known lung cancer gene signatures**

**Supporting Information Figure S1**- **The C-index results for dataset GSE68465 using gene signatures consisting of a given number of top-ranked most stage differentially expressed genes.**

**Supporting Information Figure S2** - **The performance of SDGS for survival analysis in comparison with two other five-gene GSs on patients of GSE13213 and TCGA_LUAD.**

**Supporting Information Figure S3** - **No significant differences in the survival probabilities between *EGFR*-mutant and *EGFR*-wild type patients (A), and between *TP53*-mutant and *TP53*-wild type patients (B), of the GSE13213 dataset.**

**Supporting Information Figure S4- No significant differences in the survival probabilities between low and high *PD-L1* expression patients of (A) the GSE13213 dataset, and (B) the TCGA_LUAD dataset.**

**Supporting Information Figure S5- Survival probabilities of high-risk and low-risk LUAD patients with high and low *PD-L1* expression for two different datasets by two five-gene GSs.**

**Supporting Information Figure S6**- **GSE68465 patients (all LUAD) suffered a poorer survival rate than the LUAD patients in GSE42127 and GSE14814 datasets.**

**Supporting Information Table S2- Datasets and usage in this study**

| **Dataset** | **Total number**  **of**  **patients** | **The number of ADC patients** | **usage in this study** |
| --- | --- | --- | --- |
| GSE68465 | 443 | 443 | derivation of SDGS |
|  |  | 443 | survival analysis |
|  |  | 322 | survival analysis for ACT |
| TCGA_LUAD | 475 | 475 | survival analysis |
|  |  | 475 | survival analysis for *PD-L1* expression |
| GSE13213 | 117 | 117 | survival analysis |
|  |  | 117 | survival analysis for *EGFR* status |
|  |  | 116 | survival analysis for *TP53* status |
|  |  | 117 | survival analysis for *PD-L1* expression |
| GSE14814 | 90 | 28 | survival analysis for ACT |
| GSE42127 | 176 | 133 | survival analysis for ACT |

| **Supporting Information Table S3- Overlaps of known lung cancer gene signatures** | | | | | | |  |
| --- | --- | --- | --- | --- | --- | --- | --- |
|  | **gene signature** | **Histology** | **genes** | **appear on other GS** | **%** | [**first author's affiliation**](https://www.ncbi.nlm.nih.gov/pubmed/19118056) | |
| 1 | Beer | ADC | 95 | 21 | 22.1 | Department of Surgery, University of Michigan, Ann Arbor, Michigan, USA | |
| 2 | Bhattacharjee | ADC | 151 | 39 | 25.8 | Department of Adult Oncology, Dana-Farber Cancer Institute, Harvard Medical School, 44 Binney Street, Boston, MA 02115, USA | |
| 3 | Bianchi | ADC | 10 | 2 | 20.0 | IFOM, Fondazione Istituto FIRC di Oncologia Molecolare, Milan, Italy | |
| 4 | Chen1 | NSCLC | 5 | 0 | 0.0 | National Taiwan University College of Public Health, National Taiwan University College of Medicine, Taipei, Taiwan | |
| 5 | Chen2 | other | 92 | 56 | 60.9 | Department of Biostatistics, Moffitt Cancer Center and Research Institute, Tampa, FL 33612, USA | |
| 6 | Fujiwara | NSCLC | 70 | 11 | 15.7 | Genome Science Division, Research Center for Advanced Science and Technology (RCAST), The University of Tokyo, Tokyo, Japan | |
| 7 | Kadara | ADC | 5 | 5 | 100.0 | Department of Thoracic/Head and Neck Medical Oncology, The University of Texas MD Anderson Cancer Center, Houston, Texas, USA | |
| 8 | Lu1 | ADC, SCC | 62 | 10 | 16.1 | Department of Surgery, Washington University School of Medicine, St. Louis, Missouri, USA | |
| 9 | Matsuyama | NSCLC | 169 | 10 | 5.9 | Division of Molecular Carcinogenesis, Center for Neurological Diseases and Cancer, Nagoya University Graduate School of Medicine, Nagoya, Japan | |
| 10 | Mitra | ADC | 4 | 1 | 25.0 | Division of Hematology, Department of Medicine, School of Public Health, University of Minnesota, Minneapolis, Minnesota 55455, USA | |
| 11 | Raponi_b | SCC | 45 | 7 | 15.6 | Molecular Diagnostics, Veridex LLC-a Johnson & Johnson Company, 3210 Merryfriend Row, San Diego, CA 92121, USA | |
| 12 | Parmigiani | Lung Cancer | 14 | 10 | 71.4 | Department of Oncology, Johns Hopkins University, Baltimore, Maryland 21230, USA | |
| 13 | Roepman | NSCLC | 66 | 9 | 13.6 | Agendia BV | |
| 14 | Shedden_c | ADC | 452 | 88 | 19.5 | Department of Statistics, 1085 South University, University of Michigan, Ann Arbor, Michigan 48109, USA | |
| 15 | Shedden_d | ADC | 332 | 23 | 6.9 | Department of Statistics, 1085 South University, University of Michigan, Ann Arbor, Michigan 48109, USA | |
| 16 | Sun_a | ADC | 46 | 15 | 32.6 | Department of Health Sciences Research, College of Medicine, Mayo Clinic, 200 First St SW, Rochester, MN 55905, USA | |
| 17 | Tang | ADC | 18 | 6 | 33.3 | Quantitative Biomedical Research Center, Hamon Center for Therapeutic Oncology, University of Texas Southwestern Medical Center, Dallas, TX 75390, USA | |
| 18 | Tomida1_a | NSCLC | 23 | 3 | 13.0 | Division of Molecular Oncology, Aichi Cancer Center Research Institute, Nagoya 464-8681, Japan | |
| 19 | Xie | NSCLC | 59 | 18 | 30.5 | Department of Clinical Sciences, Simmons Cancer Center, Internal Medicine, Pharmacology, and Hamon Center for Therapeutic Oncology Research, The University of Texas Southwestern Medical Center, Dallas, TX, USA | |
| 20 | SDGS | ADC | 5 | 0 | 0.0 | this work (Institute of Biomedical Sciences, Academia Sinica, Taipei, Taiwan) | |
| 21 | SDGS-associated PPI | ADC | 68 | 9 | 13.2 | this work (Institute of Biomedical Sciences, Academia Sinica, Taipei, Taiwan) | |

This list was compiled by Tang et al. (ref. no. 9 in main text) except for SDSG and SDSG’s protein-protein interaction partners, which are from the present study. Those shaded in the same color are from the same research group.


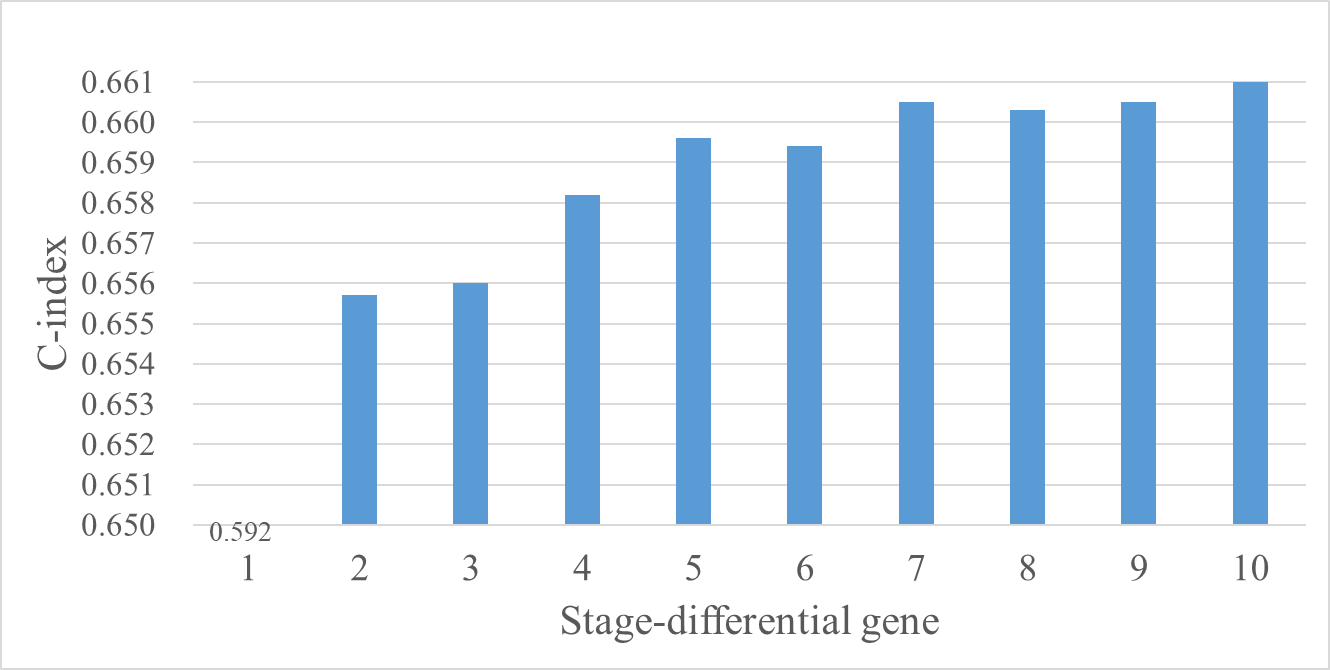


*

number of top-ranked most stage-differentially expressed genes

**Supporting Information Figure S1- The C-index results for dataset GSE68465 using gene signatures consisting of a given number of top-ranked most stage differentially expressed genes.** *: SDGS, the gene signature used in this study.


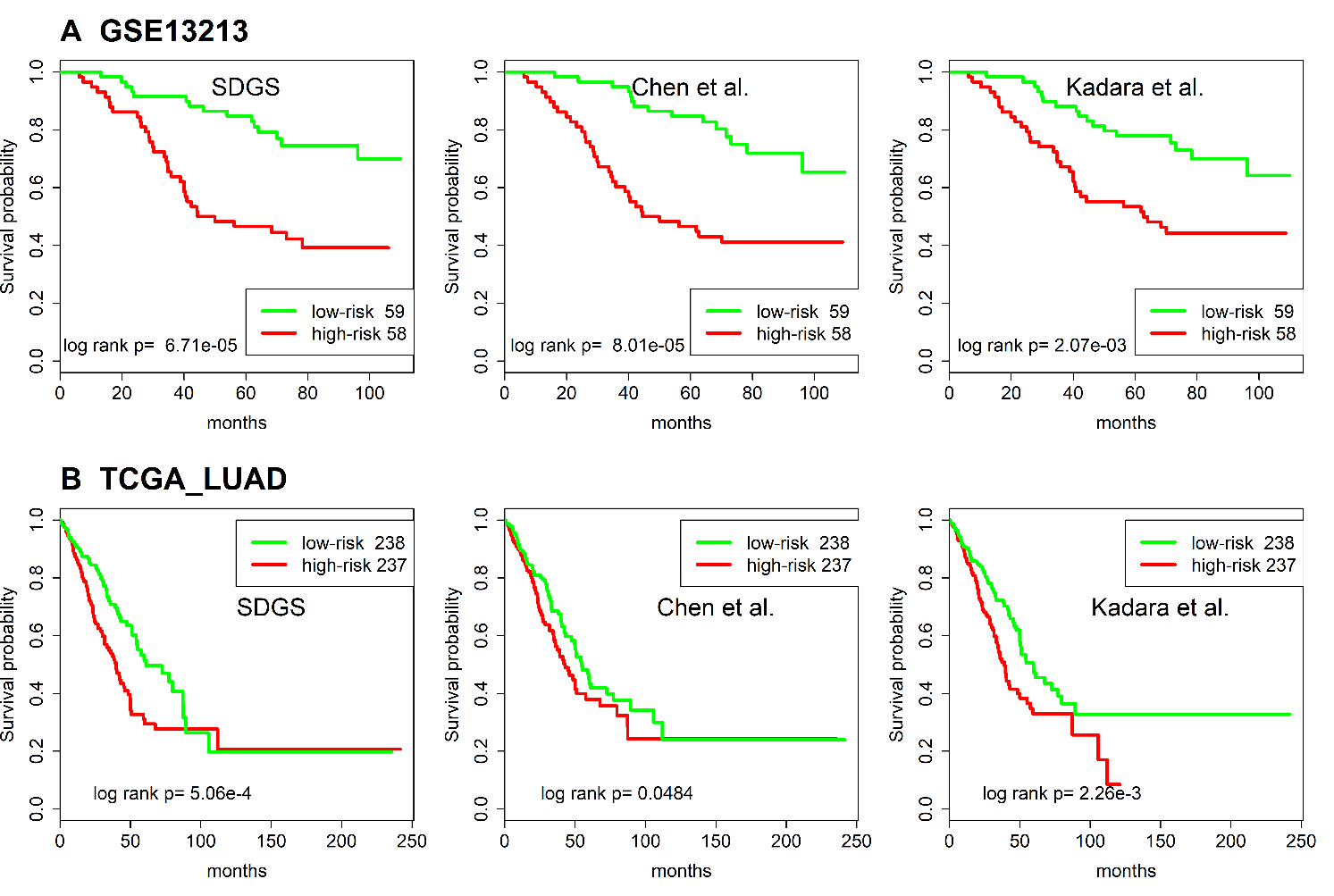


**Supporting Information Figure S2- The performance of SDGS for survival analysis in comparison with two other five-gene GSs on patients of GSE13213 and TCGA_LUAD.**

Kaplan-Meier survival curves for low-risk (green) and high-risk (red) LUAD patients of dataset (A) GSE13213 and (B) TCGA_LUAD. The log-rank p values were calculated to compare the survival differences between the low-risk and the high-risk groups of patients.

**
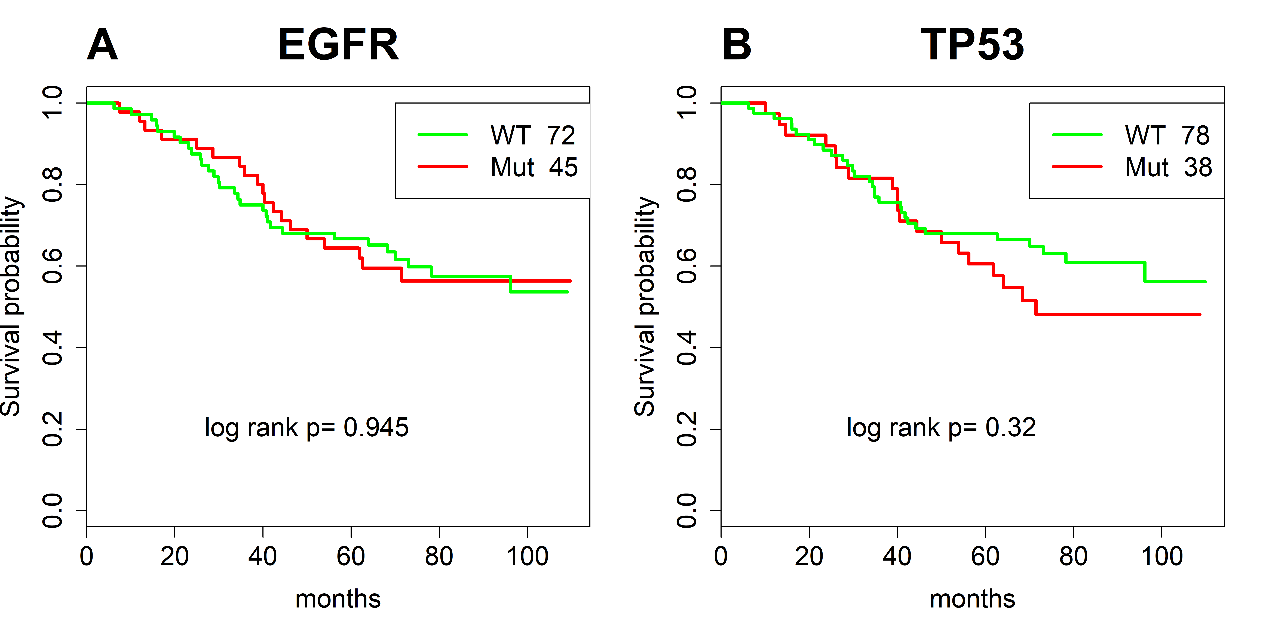
**

**Supporting Information Figure S3- No significant differences in the survival probabilities between *EGFR*-mutant and *EGFR*-wild type patients (A), and between *TP53*-mutant and *TP53*-wild type patients (B), of the GSE13213 dataset.**

**A B**

**
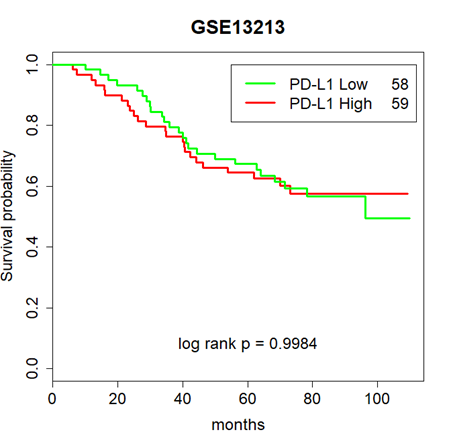

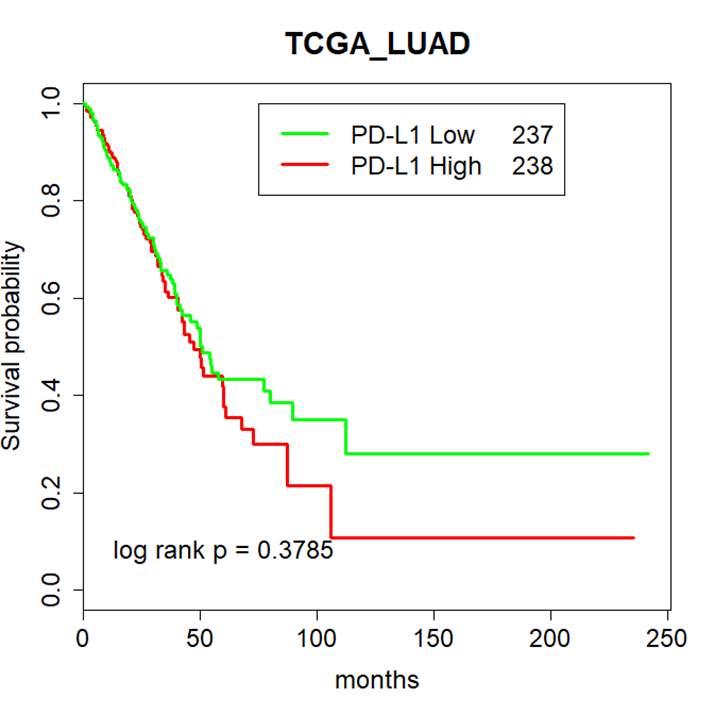
**

**Supporting Information Figure S4- No significant differences in the survival probabilities between low and high PD-L1 expression patients of (A) the GSE13213 dataset, and (B) the TCGA_LUAD dataset.**


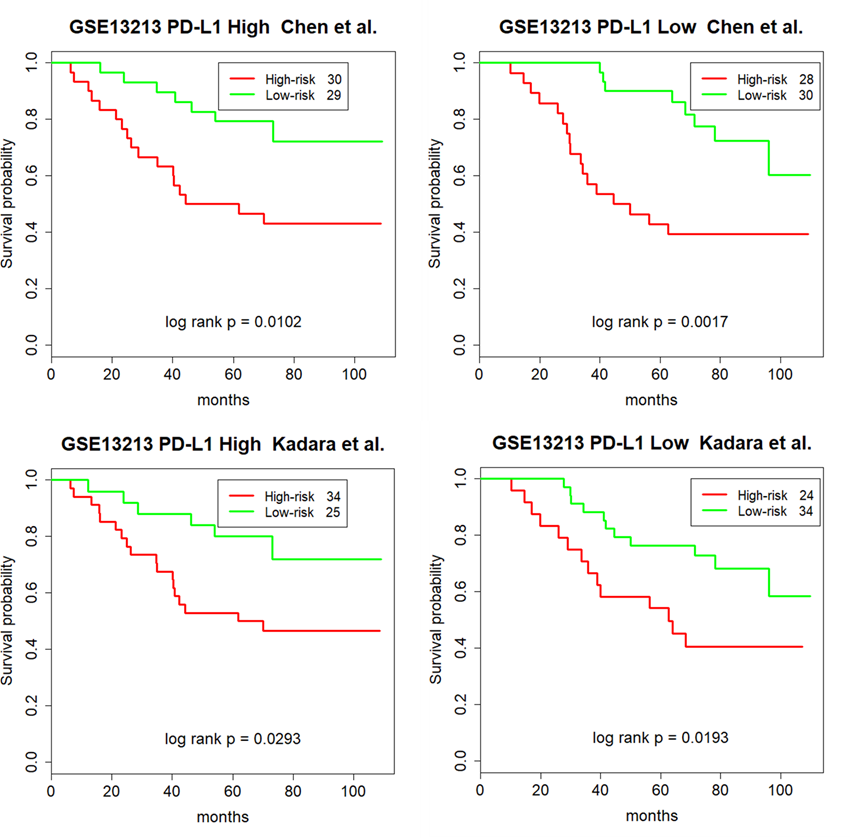
**A**


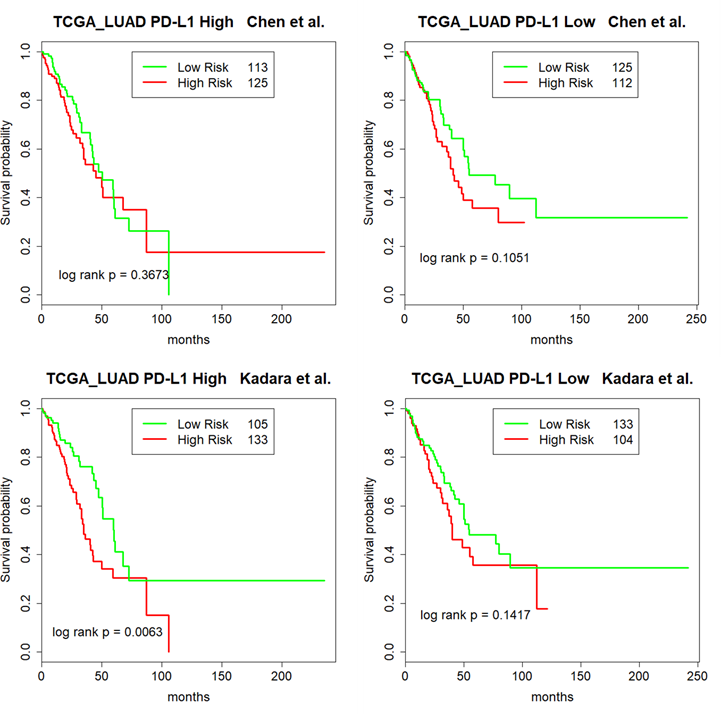
**B**

**Supporting Information Figure S5- Survival probabilities of high-risk and low-risk LUAD patients with high and low *PD-L1* expression for two different datasets by two five-gene GSs.**

Same as in **Figures 3** by the respective Cox model of the two five-gene GSs (Chen et al. and Kadara et al.) compared to SDGS but for high and low *PD-L1* expression patient groups. (A) Results for dataset GSE13213. (B) Results for dataset TCGA_LUAD.


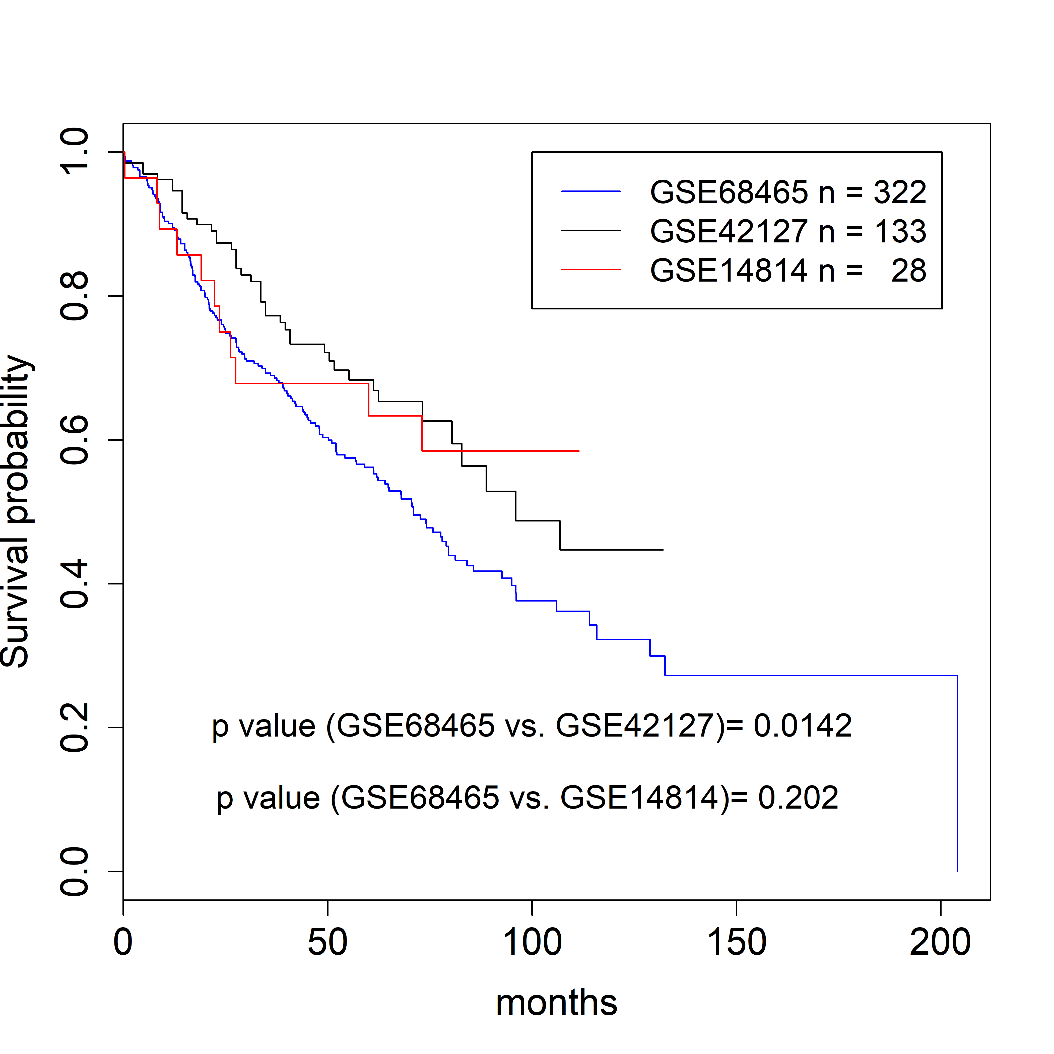
**Supporting Information Figure S6- GSE68465 patients (all LUAD) suffered a poorer survival rate than the LUAD patients in GSE42127 and GSE14814 datasets.**
